# Lp(a)-cholesterol is associated with HDL-cholesterol in overweight and obese African American children and is not an independent risk factor for CVD

**DOI:** 10.1186/1475-2840-11-10

**Published:** 2012-01-27

**Authors:** Sushma Sharma, Jayshree Merchant, Sharon E Fleming

**Affiliations:** 1 Dr Robert C and Veronica Atkins Center for Weight and Health, University of California, Berkeley, CA 94720-3104, USA; 2 Department of Nutritional Sciences and Toxicology, University of California, Berkeley, CA 94720-3104, USA; 3 Children's Hospital Oakland Research Institute, Oakland, CA 94609, USA

**Keywords:** Lipoprotein, Lp(a), LDL, HDL, TAG, TC, BMIz, Waist circumference, Obesity, CVD, Atherosclerosis

## Abstract

**Background:**

The role of Lipoprotein (a) cholesterol {Lp(a)-C}as an additional and/or independent risk factor for cardiovascular disease (CVD) is not clear. We evaluated the associations between Lp(a)-C and other CVD risk factors including plasma lipoprotein concentrations and body fatness in overweight and obese African American children.

**Methods:**

A cross-sectional analysis was carried out using data from a sample of 121 African American children aged 9-11 years with Body Mass Index (BMI)'s greater than the 85th percentile. Body height, weight and waist circumference (WC) were measured. Fasting plasma concentrations of Lp(a)-C, Total cholesterol (TC), High density lipoprotein cholesterol (HDL-C), Very low density lipoprotein cholesterol (VLDL-C), Intermediate density lipoprotein cholesterol (IDL-C), Low density lipoprotein cholesterol (LDL-C), and Triacylglycerides (TAG) were analyzed using the vertical auto profile (VAP) cholesterol method.

**Results:**

After adjusting for child age, gender, and pubertal status, Lp(a)-C was positively associated with both HDL-C and TC, and negatively associated with VLDL-C and TAG. Including BMIz and WC as additional covariates did not alter the direction of the relationships between Lp(a)-C and the other lipoproteins. Finally, after adjusting for the other plasma lipoproteins, Lp(a)-C remained strongly associated with HDL-C, whereas the associations of Lp(a)-C with the other lipoproteins were not significant when HDL-C was simultaneously included in the regression models.

**Conclusions:**

Lp(a)-C was positively associated with HDL-C and this association is not influenced by other lipoprotein subclasses or by the degree of obesity. We conclude that Lp(a) cholesterol is not an independent risk factor for CVD in African American children.

## Background

Lipoprotein (a) particles, Lp(a), were first described by Berg in 1963 [1], and are a genetic variant of low-density lipoprotein particles linked via apoB-100 to apolipoprotein(a) [2]. Since being identified, numerous studies have reported that high plasma Lp(a) concentrations are associated with atherosclerotic/thrombotic disease, as comprehensively reviewed by others [3].

Despite these reports, there have been conflicting results from prospective studies that evaluated Lp(a) as an independent risk factor for cardiovascular disease (CVD). Although the majority of studies, performed in primarily Caucasian populations, have found Lp(a) concentrations to be an independent risk factor for CVD, studies that have evaluated these relationships in African Americans have been less consistent. For example, in the biracial cohort ARIC (Atherosclerosis Risk in Communities) study of 15, 800 individuals, Lp(a) lipoprotein levels were reported to be positively associated with CVD as well as with preclinical atherosclerosis in both Black and White adults [4,5]. In a different study, however, high Lp(a) concentrations were reported not to be associated with increased risk of CVD in African Americans adults [6]. When compared to other races and ethnicities, African American adults and children have been shown to have higher plasma concentrations of Lp(a). These, among other findings, have led researchers to conclude that Lp(a) levels are regulated by both genetic and non-genetic factors [7], suggesting that a single Lp(a) cutoff would not confer the same CVD risk in African American and White populations [8].

In the U.S., plasma lipoprotein concentrations and blood pressures have been used as predictors for CVD screening, with cutoffs based on American Heart Association guidelines [9]. Individuals with higher than recommended concentrations of Total Cholesterol (TC), Low density lipoprotein cholesterol (LDL-C), Very low density lipoprotein cholesterol (VLDL-C), Intermediate density lipoprotein cholesterol (IDL-C) or Triglycerides (TAG), and lower High density lipoprotein cholesterol (HDL-C) would be considered at risk of CVD. To determine whether Lp(a) confers additional and independent risk, several studies have investigated the relationship of Lp(a) to these intermediate markers of CVD risk. Lp(a) has been reported to be positively associated with TC in Turkish adults [10] and high-school adolescents [11]; and Lp(a) and LDL-C have been reported to be positively associated in White adults [12,13], Australian Aboriginal children [14] and Taiwanese boys [15].

Lp(a) has been found also to be associated with intermediate markers of CVD risk including obesity and other lipoproteins in African Americans. Lp(a) was reported to be positively associated with TC in African American women [16] and African American 12-19 year old males [17]. Also, Lp(a) and LDL-C have been reported to be positively associated in African American men [13] and women [13,16]. Some unexpected associations have been reported. For example, Giger et al. [16] reported that Lp(a) was positively associated with HDL-C and negatively associated with TAG, VLDL-C and obesity in premenopausal African American women; and Randall et al [17] reported a negative association between Lp(a) and TAG in obese American Americans aged 12-75 years.

To the best of our knowledge, associations between Lp(a) and intermediate markers of CVD risk have not previously been reported for young African American children. Because of an epidemic increase in childhood obesity and a subsequent increase in risk for CVD, it has been recommended that children and adolescents should be screened for lipid profiles even at a young age [18,19]. Therefore, knowledge regarding the association between Lp(a)-C and markers of CVD risk in overweight and obese African American children would provide crucial information regarding choice of markers for CVD risk determination in at risk children, and others have determined that it is essential to evaluate pediatric cohorts individually due to heterogeneity [20]. The current study represents a secondary analysis of data generated from a community-based, type 2 diabetes prevention program for inner city, overweight and obese, African American children [21]. In this analysis, we aimed to evaluate the associations of Lp(a)-C with other cardiovascular disease risk factors including lipoproteins (TC, HDL-C, LDL-C, VLDL-C, TAG) and body fatness (BMIz and waist circumference) in this sample of overweight and obese, young African American children.

## Methods

### Study participants

A complete set of data for this cross-sectional analysis were available for a convenience sample of 121 (56 boys and 65 girls) African-American children who were part of a community-based lifestyle modification program that aimed to reduce the risk for type 2 diabetes mellitus as described more fully elsewhere [21]. Study participants were recruited by distributing pamphlets at local recreational sites and schools in inner-city Oakland, CA. Recruitment targeted children with at least one African American parent. Exclusion criteria included the following: BMI's below the 85th percentile when matched for age and gender; 8 years of age or younger; 12 years of age or older; fasting glucose ≥ 120 mg/dl; any known metabolic disease; or taking medications known to affect the study outcomes. Parental informed consent was obtained from all subjects, and all protocols were approved by the institutional review boards at the University of California Berkeley and San Francisco. More than 95% of the children enrolled in this study lived in inner-city Oakland, CA.

### Anthropometric measurements

Body weight and height were measured to the nearest 0.1 kg and 0.1 cm using a digital electronic scale (BWB 800, Tanita, Japan), and a portable stadiometer, respectively. Body mass index (BMI), BMI percentiles and BMI z-scores were generated using an age and gender specific CDC calculator program http://www.cdc.gov/nccdphp/dnpa/growthcharts/resources/sas.htm. Using a plastic, non-elastic measuring tape, waist circumference (WC) was measured just above the iliac crest with the child in the standing position and hip circumference was measured at the widest point above the greater trocanthers. Measurements were taken twice and if agreement between repeats exceeded 0.4 cm, a third measurement was taken and the mean calculated using the closest two values.

### Biochemical measurements

After a 12-h overnight fast, participants reported to the Children's Hospital and Research Center Oakland where their blood was drawn. Plasma lipids were measured by a comprehensive lipoprotein analysis performed by a commercial lab (LabCorp). Using the vertical auto profile (VAP) cholesterol method, a modified density gradient centrifugation technique, concentrations of the following components were determined on a single sample: TC, HDL-C, VLDL-C, IDL-C, LDL-C and TAG [22,23].

### Pubertal stage assessment

All participants were asked to report, after an overnight fast of at least 12 h, at the Children's Hospital and Research Center in Oakland, CA for blood sample collection. Pubertal development was assessed by measurements of serum luteinizing hormones (LH) in boys, and estradiol and LH in girls. Children were classified into stages 1 through 5 using literature-derived values and with cutoffs previously reported [24].

### Statistical analyses

Initially, data for 124 participants were included in the analysis. Values for several of the plasma lipoproteins were significantly skewed, and tests for non-normality remained significant even following log transformation. Using Dixon's test for outliers, data for three children were excluded to avoid unintended bias of associations in this analysis. Thus, data for 121 participants were included in the final analysis. Gender differences for anthropometric and lipoprotein characteristics were assessed using independent two-tailed t-tests following Levene's test for equality of variances. Pearson's correlations were used to determine bivariate associations. To evaluate the primary study objectives, hierarchical multiple linear regression analyses were used, with Lp(a)-C as the dependent variable. Other plasma lipoproteins were included as independent variables and models were adjusted as indicated for child age, gender, pubertal stage and body fatness. Socioeconomic status of the family was evaluated as a potential covariate but was not included in the final models since it was found to have r < 0.20 and to not be significantly related to dependent and independent variables. Statistical procedures were performed using SPSS for Windows version 18.0 PASW (SPSS, IBM Corp.). Results with *p *< 0.05 were defined to be statistically significant.

## Results

Plasma lipoprotein concentrations were considered to be in the normal range for many, but not all, of the children in this sample. Using reference interval cutoffs, less than 5% of the children had values outside the normal range for IDL-C, VLDL-C and TAG; 10-20% fell outside the normal range for TC, HDL-C and LDL-C; and nearly 25% fell outside the normal range for Lp(a)-C (Table 1). Girls in this sample of overweight and obese African American children were at a more advance pubertal stage than boys, and had significantly higher body fatness assessed by BMIz and WC values (Table 2). Differences in lipoprotein concentrations did not differ by gender. When participants were divided into three BMI percentile subgroups (< 95th, 95.001-99th, > 99th), differences among subgroups were statistically significant for concentrations of HDL-C, Lp(a)-C and TAG (Table 3).

**Table 1:** Standardized reference cutoffs for plasma lipoprotein concentrations and proportion of child participants at risk of cardiovascular disease (n = 121)

	Reference Cutoff^1^, mg/dl	At risk,% of sample
TC	> 200	17.4

HDL-C	≤ 40	12.4

VLDL-C	> 30	1.7

IDL-C	> 20	3.3

LDL-C	> 130	14.0

TAG	> 150	1.7

Lp(a)-C	> 10	24.8

^1^Reference cutoff intervals taken from LabCorp standards for VAP Cholesterol analyses https://www.labcorp.com/pdf/VAP_test_flyer.pdf

*Abbreviations*: Total cholesterol: TC, High density lipoprotein cholesterol: HDL-C, Very low density lipoprotein cholesterol: VLDL-C, Intermediate density lipoprotein cholesterol: IDL-C, Low density lipoprotein cholesterol: LDL-C, Triglycerides: TAG, Lp(a) cholesterol: Lp(a)-C

**Table 2:** Characteristics of participating African-American boys and girls

	Boys	Girls	*p*-value^1^
Sample size, n	56	65	

**Anthropometrics**	**Mean (SEM)**		
		

Age (years)	10.7 (0.1)	10.6 (0.1)	0.575

Pubertal stage	2.4 (0.2)	3.5 (0.1)	**< 0.001**

Height (cm)	148.1 (0.02)	150.8 (0.01)	0.248

Weight (kg)	62.5 (2.6)	69.1 (2.3)	0.057

BMI-z score	2.0 (0.07)	2.2 (0.05)	**0.050**

WC (cm)	87.0 (2.1)	93.0 (1.8)	**0.032**

**Plasma lipoproteins, mg/dl**			

TC	168.0 (3.6)	168.3 (4.6)	0.955

HDL-C	56.2 (1.6)	52.0 (1.4)	0.052

VLDL-C	15.9 (0.6)	16.1 (0.4)	0.736

IDL-C	9.1 (0.6)	9.4 (0.6)	0.724

LDL-C	95.9 (3.2)	100.4 (4.0)	0.371

TAG	68.4 (4.4)	75.1 (3.1)	0.202

Lp(a)-C	7.8 (0.54)	8.1(0.51)	0.699

**^1^**Statistical significance of differences determined using two-tailed t-test following Levene's test for equality of variances

*Abbreviations*: Body mass index: BMI, Body mass index z-score: BMIz, Waist circumference: WC, Total cholesterol: TC, High density lipoprotein cholesterol: HDL-C, Very low density lipoprotein cholesterol: VLDL-C, Intermediate density lipoprotein cholesterol: IDL-C, Low density lipoprotein cholesterol: LDL-C, Triglycerides: TAG, Lp(a) cholesterol: Lp(a)-C

**Table 3:** Influence of BMI percentile category on characteristics of participating children

Classification BMI percentiles	At risk of overweight 85-95th	Overweight & Obese		
		
		95.001-99 th	> 99 th	*p*-value^1^
Sample size, n	24	48	49	

**Anthropometrics**	**Mean (SEM)**			

Age (years)	10.5 (0.2)	10.8 (0.1)	10.7 (0.1)	0.412

Pubertal stage	2.75 (0.3)	3.14(0.2)	2.95 (0.2)	0.574

Height (cm)	147.3 (1.8)	150.2 (1.3)	152.1 (1.3)	0.118

Weight (kg)	46.6 (1.5)	61.0 (1.4)	80.5 (2.6)	**< 0.001**

BMI-z score	1.34 (0.4)	2.03 (0.03)	2.54 (0.02)	**< 0.001**

WC (cm)	71.8 (1.4)	86.9 (1.19)	102.4 (1.8)	**< 0.001**

**Plasma lipoproteins, mg/dl**				

TC	177.7 (7.0)	161.1 (4.1)	170.5 (4.9)	0.100

HDL-C	62.2^b ^(2.8)	53.0^a ^(1.4)	50.8^a ^(1.5)	**< 0.001**

VLDL-C	15.6 (0.9)	15.2 (0.5)	16.9 (0.5)	0.099

IDL-C	9.38 (1.0)	8.33 (0.6)	10.1 (0.6)	0.172

LDL-C	100.1 (6.2)	92.9 (3.6)	102.7 (4.3)	0.227

TAG	59.5^a ^(4.5)	68.5^a, b ^(3.8)	81.5^b ^(4.5)	**0.005**

Lp(a)-C	10.7^b ^(0.9)	7.75^a ^(0.5)	6.71^a ^(0.5)	**< 0.001**

**^1^**Differences determined using two-tailed t-test following Levene's test for equality of variances. Groups sharing a common superscript are not significantly different from each other using Tukey's studentized range test at a 5% procedure-wise error rate

*Abbreviations*: Body mass index: BMI, Body mass index z-score: BMIz, Waist circumference: WC, Total cholesterol: TC, High density lipoprotein cholesterol: HDL-C, Very low density lipoprotein cholesterol: VLDL-C, Intermediate density lipoprotein cholesterol: IDL-C, Low density lipoprotein cholesterol: LDL-C, Triglycerides: TAG, Lp(a)ccholesterol: Lp(a)-C

Body fatness, assessed using either BMIz scores and WC, was significantly and negatively correlated with HDL-C and Lp(a)-C, and significantly but positively associated with TAG (Table 4). TC was positively associated with all concentrations of all lipoprotein subclasses and, among the subclasses, HDL-C was negatively associated with VLDL-C and TAG; and VLDL-C, IDL-C and LDL-C were intercorrelated. Lp(a)-C was positively correlated with TC and HDL-C, and negatively correlated with VLDL-C and TAG.

**Table 4:** Pearson correlation coefficients and significance^1 ^among measure of obesity and plasma lipoprotein concentrations (n = 121)

	WC	TC	HDL-C	VLDL-C	IDL-C	LDL-C	TAG	Lp(a)-C
BMIz	0.801***	-0.019	-0.348***	0.212*	0.134	0.090	0.357***	-0.394***

WC		-0.107	-0.476***	0.187*	0.104	0.050	0.332***	-0.337***

TC			0.384***	0.310***	0.562***	0.934***	0.228*	0.219*

HDL-C				-0.224*	-0.117	0.051	-0.285**	0.462***

VLDL-C					0.803***	0.306***	0.869***	-0.254**

IDL-C						0.574***	0.699***	-0.155

LDL-C							0.258**	0.097

TAG								-0.283**

^1^Significance: * for *p *< 0.05, ** for *p *< 0.01, *** for *p *< 0.001

*Abbreviations*: Body mass index: BMI, Body mass index z-score: BMIz, Waist circumference: WC, Total cholesterol: TC, High density lipoprotein cholesterol: HDL-C, Very low density lipoprotein cholesterol: VLDL-C, Intermediate density lipoprotein cholesterol: IDL-C, Low density lipoprotein cholesterol: LDL-C, Triglycerides: TAG, Lp(a) cholesterol: Lp(a)-C

Using multiple regression analysis, and following adjustments for child age, gender and pubertal stage, Lp(a)-C remained significantly and positively associated with TC and HDL-C, and remained significantly and negatively associated with VLDL-C and TAG (Table 5). Since obesity was highly correlated with Lp(a)-C, we included WC and BMIz (separately) as additional covariates. Associations of Lp(a)-C with TC, HDL-C, VLDL-C and TAG continued to be statistically significant after these adjustments for body fatness.

**Table 5:** Relationship of other plasma lipoproteins to Lp(a)-C concentrations (n = 121)

		Independent variables^1^					
**Models**	**Other variables included in models**	**TC**	**HDL-C**	**VLDL-C**	**IDL-C**	**LDL-C**	**TAG**

		**Standardized regression coefficient with Lp(a) as dependent variable^2^**					

I	None	0.219*	0.462***	-0.254**	-0.155	0.097	-0.283**

II	Covar	0.244**	0.541***	-0.276**	-0.166	0.114	-0.307***

III	Covar + BMIz	0.235**	0.446***	-0.196*	-0.113	0.145	-0.187*

IV	Covar + WC	0.227**	0.450***	-0.200*	-0.108	0.142	-0.190*

^1^Regression analyses were performed separately for each independent lipoprotein variable. Coefficients are shown following adjustments for covariates (covar) including child sex, age, pubertal stage, group assignment and family socio-economic status

^2^Significance: * for *p *< 0.05, ** for *p *< 0.01, *** for *p *≤ 0.001

*Abbreviations*: Body mass index: BMI, Body mass index z-score: BMIz, Waist circumference: WC, Total cholesterol: TC, High density lipoprotein cholesterol: HDL-C, Very low density lipoprotein cholesterol: VLDL-C, Intermediate density lipoprotein cholesterol: IDL-C, Low density lipoprotein cholesterol: LDL-C, Triglycerides: TAG, Lp(a) cholesterol: Lp(a)-C

Since Lp(a)-C appeared to be consistently and more strongly related to HDL-C than to the other lipoprotein subclasses (Table 5), a final analysis was performed to determine if the association between Lp(a)-C and HDL-C was accounted for by other plasma lipoproteins since inter-correlations were observed among the lipoprotein subclasses (Table 5). The strong relationship between HDL-C and Lp(a)-C was not explained by its association with other lipoprotein subclasses since, after adjusting for each subclass in a separate regression model, Lp(a)-C remained highly related to HDL-C whereas associations of Lp(a)-C with other lipoprotein subclasses were no longer statistically significant (Table 6).

**Table 6:** Relationship of plasma concentrations of Lp(a)-C to HDL-C before and after adjustments for other plasma lipoproteins (n = 121)

	Independent variables					
**Models^1^**	**HDL-C**	**TC**	**VLDL-C**	**IDL-C**	**LDL-C**	**TAG**

	**Standardized regression coefficients with Lp(a) as dependent variable^2^**					

IV	0.450***					

V	0.411***	0.093				

VI	0.424***		-0.135			

VII	0.442***			-0.070		

VIII	0.440***				0.114	

IX	0.428***					-0.121

^1^Regression analyses were performed for models that included the covariates (child age, gender, pubertal stage and waist circumference), and with HDL-C entered alone (model IV) or with one of the other independent lipoprotein variables of interest (models V-IX)

^2^Significance: *** for p ≤ 0.001

*Abbreviations*: Total cholesterol: TC, High density lipoprotein cholesterol: HDL-C, Very low density lipoprotein cholesterol: VLDL-C, Intermediate density lipoprotein cholesterol: IDL-C, Low density lipoprotein cholesterol: LDL-C, Triglycerides: TAG, Lp(a) cholesterol: Lp(a)-C

Finally, we evaluated the frequency with which children in our cohort were simultaneously "at risk" of CVD using the cutoffs provided in Table 1. None of the children in the "at risk" categories using cutoffs for HDL-C, VLDL-C, IDL-C or TAG were in the "at risk" category for Lp(a) (data not shown). Of the children in the "at risk" categories using the cutoffs for TC or LDL-C, only 1/3 were in the "at risk" category for Lp(a).

## Discussion

To our knowledge this is one of the first studies to report a strong positive association between Lp(a)-C and HDL-C in overweight and obese African American children. Positive correlations between Lp(a)-C and HDL-C concentrations have been reported previously in premenopausal African American women [16], and our results show that this relationship can be observed prior to adulthood. Although, others have reported no association between Lp(a)-C and HDL-C in normal weight girls [25], our results expand on the current literature by showing that the strong positive association between Lp(a)-C and HDL-C in overweight and obese African American children included in our study. This association remained following adjustments for body fatness and following adjustments for other plasma lipoprotein subclasses.

The strong association of Lp(a)-C with HDL-C observed in our study suggests that increasing Lp(a)-C concentrations may not be reliably used as a marker of CAD risk in this population. Instead, our results agree with those of others [17] who observed that a therapeutic lifestyle modification program administered to obese African Americans aged 12-75 years caused levels of Lp (a) and HDL-C to increase and caused LDL-C, TC, TG and BMI to decrease. Those authors reasoned that, since previous studies with therapeutic life style change have shown a favorable impact on cardio vascular health, their results, like ours, did not support the established understanding that elevations in Lp(a) would be atherogenic and harmful. Thus, our results in overweight and obese African American children are in line with the suggestion, made by others [6], that increasing plasma concentration of Lp(a) is not an independent risk factor for CVD in African American adults.

Lp(a) is known to consist of an LDL-like particle covalently linked via an apo-B100 molecule to apolipoprotein (a), and these particles are variable in its size and cholesterol content. Lp(a) and LDL have been reported to be positively correlated in African American females [16,25] and in Taiwanese boys [15] and white girls [11]. Relationships were reported not to be significant, however, in Taiwanese girls [15], white boys, black boys and black girls [11]. In our population, Lp(a)-C and LDL-C concentrations also were not found to be significantly associated even after adjusting for child characteristics including degree of obesity. This lack of association is unlikely to be due to the analytical procedure used since Lp(a)-C, measured using the VAP-C technique as we have done, has been reported to be highly correlated to Lp(a) mass measured using ELISA [23].

In our cohort of overweight African American children, Lp(a) was significantly and negatively associated with TAG concentrations prior to an following adjustment for obesity. Others have reported also a negative association between LP(a) and TAG in American Americans aged 12-75 [17], African American women [16], Turkish men and women [10], and Taiwanese girls [15]. Although our results agree with those of others, the association was no longer significant following adjustment for HDL-C. This suggests that HDL-C accounts for the association between Lp(a) and TAG observed by us and others.

Others have previously reported that African American children and adolescents have higher levels of Lp(a) and HDL than their non-Hispanic white peers [11,25,26]. In our cohort, nearly 25% of children had Lp(a)-C concentrations that placed them in the "at risk" category whereas only 12% had HDL-C concentrations that placed them in the "at risk" category. Of interest, is our observation that, of the 15 individual children in our cohort with low "at risk" HDL-C, none had "at risk" Lp(a)-C concentrations. Thus, the strong positive association we observed between Lp(a)-C and HDL-C suggests it is unlikely that both of these factors would serve as a reliable marker for risk of CVD in overweight and obese African American children.

In our population, Lp(a)-C concentrations were negatively related to body fatness assessed using either BMIz or WC, and children between the 85th and 95th BMI percentile had significantly higher Lp(a)-C concentrations than children with BMIs above the 95th percentile. Previously, Lp(a)-C concentrations were reported to be negatively correlated with body weight and waist-to-hip ratio in African American women [16], although no association was found between Lp(a) levels and obesity in non-African American children or adults [15,27]. Because Lp(a)-C was correlated with obesity in our population, BMIz or WC were included as covariates in our multiple regression models. Our results show, however, that the positive association between Lp(a)-C and HDL-C was independent of the degree of obesity in these children.

This pilot analysis includes restriction to low-income, inner-city, African American children and exclusion of children with BMI's less than the 85th percentile when matched for age and gender. These limitations preclude extrapolation to the wider population of children of different races, ages and socioeconomic backgrounds, and comparisons with lower BMI children. This is a cross-sectional analysis of data, precluding a cause and effect relationship. Lipoprotein subclasses were analyzed using the VAP-cholesterol technique, precluding extrapolation to results based on other analytical techniques that quantify numbers or size of particles or associated protein.

## Conclusions

Lp(a)-C was positively associated with HDL-C in overweight and obese African American children and this association remained following adjustments for other lipoprotein subclasses and degree of obesity. Based on our study, we propose that Lp(a) is not associated with lipoprotein recognized as markers of increased risk of CVD in overweight and obese African American children. Further studies will be needed to explain the basis for the association of Lp(a)-C and HDL-C in these children.

## Abbreviations

Lp(a): Lipoprotein (a); Lp(a)-C: Lp(a) cholesterol; LDL-C: Low density lipoprotein cholesterol; HDL-C: High density lipoprotein cholesterol; IDL-C: Intermediate density lipoprotein cholesterol; VLDL-C: Very low density lipoprotein cholesterol;TAG: Triglycerides; TC: Total cholesterol; BMI: Body mass index; BMIz: Body mass index z-score; WC: Waist circumference; CVD: Cardiovascular disease.

## Competing interests

The authors declare that they have no competing interests.

## Authors' contributions

Contributor's list: Dr.S contributed in statistical analysis, prepared the manuscript and submission. Ms. Merchant provided analytical support and expertise in interpretation of data. Prof.F was the principal investigator of the study. She supervised the design and execution of the study and manuscript. All authors read and approved the final manuscript.
